# Design, Modeling, and Experimental Validation of a Bio-Inspired Rigid–Flexible Continuum Robot Driven by Flexible Shaft Tension–Torsion Synergy

**DOI:** 10.3390/biomimetics10050301

**Published:** 2025-05-08

**Authors:** Jiaxiang Dong, Quanquan Liu, Peng Li, Chunbao Wang, Xuezhi Zhao, Xiping Hu

**Affiliations:** 1 School of Mechanical and Automotive Engineering, South China University of Technology, Guangzhou 510640, China; 202110183011@mail.scut.edu.cn (J.D.); mezhaoxz@scut.edu.cn (X.Z.); 2 Guangdong-Hong Kong-Macao Joint Laboratory, Artificial Intelligence Research Institute, Shenzhen MSU-BIT University, Shenzhen 518172, China; huxp@smbu.edu.cn; 3 School of Mechanical and Electrical Engineering and Automation, Harbin Institute of Technology (Shenzhen), Shenzhen 518000, China; 4 School of Mechanical and Electrical Engineering, Guangdong University of Science and Technology, Dongguan 523083, China; chunbaowang@163.com

**Keywords:** octopus arm inspired actuation, continuum robot, rigid–flexible hybrid robot, tensile–torsional synergistic actuation, kinetostatic modeling, flexible shafts

## Abstract

This paper presents a bio-inspired rigid–flexible continuum robot driven by flexible shaft tension–torsion synergy, tackling the trade-off between actuation complexity and flexibility in continuum robots. Inspired by the muscular arrangement of octopus arms, enabling versatile multi-degree-of-freedom (DoF) movements, the robot achieves 6-DoF motion and 1-DoF gripper opening and closing movement with only six flexible shafts, simplifying actuation while boosting dexterity. A comprehensive kinetostatic model, grounded in Cosserat rod theory, is developed; this model explicitly incorporates the coupling between the spinal rods and flexible shafts, the distributed gravitational effects of spacer disks, and friction within the guide tubes. Experimental validation using a physical prototype reveals that accounting for spacer disk gravity diminishes the maximum shape prediction error from 20.56% to 0.60% relative to the robot’s total length. Furthermore, shape perception experiments under no-load and 200 g load conditions show average errors of less than 2.01% and 2.61%, respectively. Performance assessments of the distal rigid joint showcased significant dexterity, including a 53° grasping range, 360° continuous rotation, and a pitching range from −40° to +45°. Successful obstacle avoidance and long-distance target reaching experiments further demonstrate the robot’s effectiveness, highlighting its potential for applications in medical and industrial fields.

## 1. Introduction

Nature has long inspired the development of robotic systems capable of navigating complex and confined environments, as exemplified by the sinuous motion of snakes [1], the dexterous flexibility of octopus arms [2], and the adaptable compliance of vertebrate spinal structures [3,4]. In scenarios such as minimally invasive surgery (MIS) and industrial inspections within confined spaces, bio-inspired continuum robots exhibit significant application potential owing to their unique structural compliance and high flexibility [5,6]. Conversely, traditional rigid robotic arms often encounter limitations related to joint’s DoFs and structural dimensions, hindering safe and precise operations in such complex, constrained environments [7]. By mimicking biological systems, continuum robots can seamlessly adapt to complex pathways, making them ideal for delicate tasks in human cavities—such as the digestive, respiratory, or urogenital tracts [8,9]. Drawing from biological analogs, these robots offer a highly promising technological paradigm for tasks requiring high dexterity and adaptability.

The selection of an actuation mechanism critically determines the performance envelope of bio-inspired continuum robots. Prevalent actuation methods include tendon-driven [10,11], pneumatically-driven [12], shape memory alloy (SMA)-driven [13], and magnetically-driven [14]. While pneumatic drives facilitate large deformations, they suffer from response hysteresis and necessitate bulky systems. SMA drives are constrained by thermodynamic hysteresis and power density limitations. Magnetic drives, although circumventing wear issues inherent in traditional mechanical transmissions via non-contact methods, face challenges concerning energy efficiency and environmental sensitivity. In contrast, tendon-driven stands out due to its rapid response, high energy density, and the ease of configuring drive units remotely from the robot body, making them particularly suitable for bio-inspired designs. However, existing tendon-driven systems typically employ the “pure traction” mode, using multiple sets of parallel tendons to coordinately control the bending of flexible segments [15,16]. However, such designs confront two primary challenges: first, achieving multi-degree-of-freedom control necessitates the configuration of numerous tendons, substantially increasing mechanism complexity; second, the end-effector lacks independent torque output capability, restricting its utility in tasks requiring gripping or twisting actions. To address the limitations of traditional tendon-driven systems, recent studies have begun exploring the potential of flexible shaft actuation, which provides a new design paradigm for continuum robots through the synergistic effects of stretching and twisting. Tan et al. [17] proposed an innovative soft actuator that utilizes flexible shafts to achieve coordinated actuation with three degrees of freedom: stretching, compression, and twisting. Similarly, Liu et al. [18] developed a soft robotic gripper driven by three flexible shafts controlling three soft fingers, enabling simultaneous grasping and bottle cap manipulation. However, both studies primarily focus on the performance characterization of individual soft actuators, and the torque transmission capability of the flexible shafts is limited by the softness of the materials. Moreover, neither sufficiently integrates the power transmission function of rigid end joints, which restricts their application potential in rigid-flexible hybrid systems.

To overcome these limitations, rigid–flexible hybrid mechanisms have garnered increasing research attention in recent years. These designs seek to synergize the adaptability of flexible segments with the load-bearing capacity and operational precision of rigid segments [19]. Prior studies have explored various implementations and applications of rigid–flexible hybrid designs. For instance, Liu et al. [20] proposed combining flexible LCE-MXene composite materials with rigid springs to realize a hybrid structure, yet this approach is still limited in output force and control accuracy due to external power supply and material nonlinearity. Menciassi et al. [21] integrated two identical flexible actuators within a rigid mechanical frame, utilizing an antagonistic configuration to balance high compliance with substantial output force; however, its dependence on external pneumatic supply and manufacturing process limitations restrict broader adoption. Additionally, Wang et al. [22] proposed a 6-DoF robotic system for in situ aircraft engine blade repair, integrating a 3-PRS parallel mechanism with tendon-driven flexible continuum segments. While this design balances precision positioning and posture adaptability, its auxiliary 3-PRS mechanism increases actuation system complexity and bulk, and the distal flexible manipulator, limited to tendon-driven actuation, achieves only three DoFs, reducing its versatility for complex tasks. In summary, while research on rigid–flexible hybrid mechanisms has achieved notable progress concerning actuation methods, DoF configurations, and application scenarios, critical challenges—such as insufficient DoFs in the distal manipulator, limited output force, and control precision challenges—remain prominent and necessitate effective solutions. This study introduces a novel flexible robot structure driven by the tensile–torsional synergistic actuation of flexible shafts. Unlike traditional tendons that solely transmit tensile forces, this design ingeniously leverages the dual functionality of the flexible shaft: serving as a medium for tensile-bending actuation of the flexible segments and concurrently transmitting torque to the distal rigid joint. This approach markedly simplifies the robot’s drive and transmission system, obviates the need for auxiliary complex drive chains for the rigid joint, and reduces structural complexity while preserving or even enhancing the robot’s overall dexterity.

Accurate mathematical modeling is the foundation for achieving shape perception and precise control in continuum robots [23,24]. Piecewise constant curvature (PCC) models are widely adopted due to their simplicity and computational efficiency [25,26]. However, the underlying constant curvature assumption limits the accuracy of these models under external loading or complex actuation coupling [27]. Approaches based on beam constraint models [28,29] and the principle of virtual power [30] can partially mitigate this limitation. Nevertheless, for a comprehensive and precise geometric and mechanical description of slender flexible structures in three-dimensional space—particularly accounting for large bending, torsion, extension, shear deformations, and their intricate nonlinear coupling effects—Cosserat rod theory offers a more fundamental and exhaustive framework [31]. The applicability of Cosserat rod theory in flexible robot modeling is further supported by [32,33]. Leveraging its advantages in capturing complex mechanical behaviors, this study formulates the kinetostatic model of the proposed bio-inspired rigid-flexible continuum robot based on Cosserat rod theory. This model not only characterizes the deformation behavior of the coupled ‘spinal rods + flexible shafts’ system within the dual flexible segments but also integrates the kinematics of the intermediate rigid connecting segment and the distal rigid joint. By incorporating distributed gravitational effects of the spacer disks and friction models within the guide tubes, this work constructs a more comprehensive kinetostatic model aimed to accurately predict the robot’s static configuration under diverse driving forces and external loads.

The main contributions of this study are summarized as follows:

1. A novel design is presented for a bio-inspired rigid–flexible continuum robot driven by flexible shaft tension and torsion synergistically, including a two-segment flexible joint driven by flexible shaft tension and a rigid joint driven by flexible shaft torsion.
2. To characterize the deformation and loading behavior of this hybrid mechanism, a kinetostatic model based on Cosserat rod theory is developed for the coupled “spinal rods + flexible shafts” system, encompassing the double-segment flexible joint, a rigid connecting segment, and the single-segment rigid joint.
3. By incorporating models for guide tube friction and the distributed gravity of the spacer disks, a comprehensive kinetostatic model of the bio-inspired rigid–flexible continuum robot is formulated. This model accounts for the effects of flexible shaft tension, the gravity of the flexible shaft, spinal rod, and spacer disk, as well as external forces/moments.
4. A physical prototype of the bio-inspired rigid–flexible continuum robot was fabricated. Experimental validations, including assessments of spacer disk mass effects, shape prediction accuracy, and the motion characteristics of the rigid joint, were performed to validate the developed kinetostatic model. The results demonstrate the model’s capability to predict robot deformation driven and controlled by flexible shaft forces, further laying the foundation for precise control of the bio-inspired rigid–flexible continuum robot.

The remainder of this paper is organized as follows: Section 2 details the system architecture and operational principles. Section 3 elaborates on the kinetostatic modeling methodology grounded in Cosserat rod theory. Section 4 presents experimental results, including guide tube parameter calibration, spacer disk gravity validation, shape perception accuracy assessment, and rigid joint motion performance evaluation. Section 5 discusses the experimental findings and their implications in detail. Finally, Section 6 summarizes the key findings and outlines potential avenues for future research.

## 2. System Description

**Figure 1:**
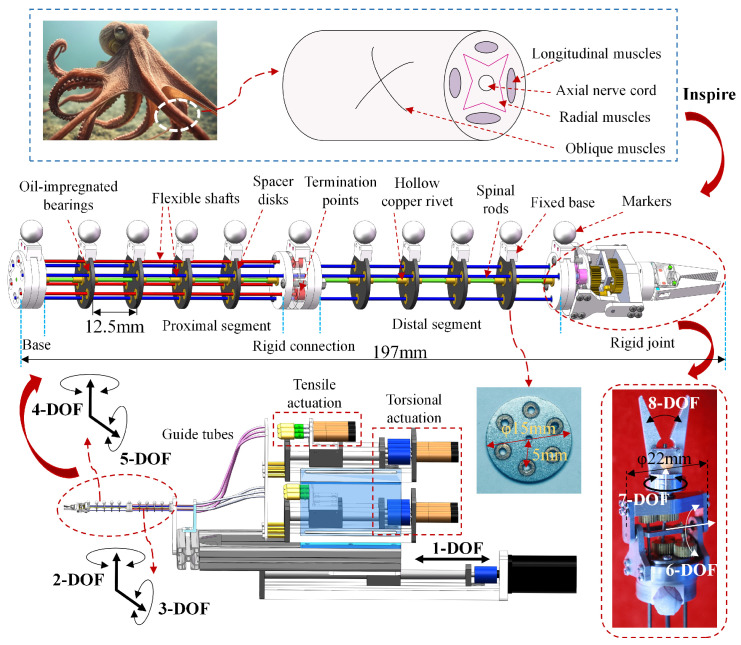
Bio-inspired rigid-flexible continuum robot structure.

The bio-inspired rigid-flexible continuum robot, shown in Figure 1, draws inspiration from the flexible, multi-DoF movements of octopus arms, as seen in natural systems capable of navigating complex environments and performing dexterous manipulation. To illustrate the biological inspiration, Figure 1 depicts a cross-section of an octopus arm, highlighting the arrangement of key muscular components: longitudinal muscles, axial nerve cord, radial muscles, and oblique muscles [34]. This robot integrates flexible and rigid joints: the flexible joints are driven by flexible shafts, with the shafts arranged along spacer disks on the central spinal rod; the rigid joints use the transmission mechanism consisting of springs and gear pairs. The central spinal rod ensures structural integrity, mimicking the supportive role of the muscular hydrostat in an octopus arm, while flexible shafts emulate the longitudinal muscles, enabling smooth, continuous bending [35]. The robot mainly consists of the proximal segment of the flexible joint, the distal segment of the flexible joint, the rigid connecting segment, the rigid joint, the specially shaped guide tubes, and the drive units. Six DC brushless motors provide tensile actuation for four orthogonal bending DOFs of the flexible joints, while other three DC brushless motors provide torsional actuation for three DOFs of the rigid joint, enabling pitch, rotation, and gripper opening/closing movements. A single DC brushless motor powers the linear reciprocating motion of the entire manipulator. The configuration and distribution of all DOFs are schematically shown in Figure 1. Six flexible shafts originate at the motor output shafts, terminate at the ends of the proximal/distal segments of the flexible joints, and are routed and guided via six specially shaped guide tubes.

To further enhance the stability of the spacer disks on the spinal rod and prevent slippage, two hollow copper rivets are fixed on each side of the spacer disk with adhesive, effectively increasing the effective bonding area between the spacer disk and the spinal rod. To address the friction issues between the flexible shaft and spacer disk [36], powder metallurgy oil-impregnated bearings are embedded at the contact points between the spacer disk and the flexible shaft. The outer ring of the bearing is press-fit into the inner hole of the spacer disk, and the flexible shaft passes through the center hole of the bearing, which has a diameter of 1.1 mm. Spacer disks with varying hole shapes and a specific stacking sequence enable rapid modular assembly and disassembly of the manipulator. Additionally, the bolted connection of the modular spacer disks also provides a solid mounting base for the rigid joints. For motion capture, reflective markers are installed on the fixed base at the same height, and the precise alignment of all reflective markers is ensured by inserting two tungsten steel rods into positioning holes on the fixed base.

## 3. Kinetostatic Model of the Bio-Inspired Rigid–Flexible Continuum Robot

This section adopts the Cosserat rod theory to perform the kinetostatic modeling of the bio-inspired rigid–flexible continuum robot. The nonlinear description allows for accurate modeling of the geometric and mechanical behavior of flexible robots with slender structures, especially for large deformations and complex loading conditions, as the theory does not rely on approximations of small displacements or slopes [37]. To reduce model complexity and facilitate numerical solutions, the following assumptions are made:

1. Based on the multiple spacer disk layout scheme, the flexible shaft is treated as a continuous structural constraint. This assumption significantly reduces friction between the flexible shaft and the structure, avoiding the need for detailed solving of the flexible shaft shape, thus simplifying the model complexity of the bio-inspired rigid–flexible continuum robot.
2. The flexible shaft is assumed to be non-retractable and to experience negligible friction with the spacer disks, an assumption justified by the use of powder metallurgy oil-impregnated bearings.
3. The spinal rod material follows a linear constitutive relationship, and its material properties are assumed to be constant along its length.
4. Since the proximal and distal segments of the flexible joints are bolted together, additional forces and moments arising at the interface due to the flexible shafts are ignored in the kinetostatic model.

### 3.1. Kinematics

The kinematics of the bio-inspired rigid–flexible continuum robot mainly includes three parts: the flexible joints, the rigid connecting segment, and the rigid joints.

#### 3.1.1. Kinematics of Flexible Joints

Based on the Cosserat rod theory [38], the shape of the spinal rod is defined as a parameterized Cartesian curve. This curve is described by $\left[R\left(s\right)\;\;p\left(s\right)\right]$, where the length parameter $s\in \left[0\;l\right]$, with *l* being the length of the spinal rod, $p\left(s\right)\in R^{3}$ is the position vector, and $R\left(s\right)$ is the orientation matrix, which belongs to the special orthogonal group $SO\left(3\right)$ of 3D space, satisfying $SO\left(3\right)=\left\{R\in R^{3\times 3}|R^{T}R=I\;\mathrm{and}\;\det\left(R\right)=1\right\}$. To combine the rotational and translational transformations of the spinal rod, we use $g\left(s\right)$ to describe the pose transformation of the rod, which belongs to the special Euclidean group $SE\left(3\right)$ in 3D space, as follows:

(1) $$g\left(s\right)=\left[\begin{array}{cc} R\left(s\right) & p\left(s\right)\\ 0_{1\times 3} & 1 \end{array}\right]$$

Based on the Lie group and Lie algebra theory, it is known that $g{\left(s\right)}^{-1}\dot{g}\left(s\right)$ belongs to the Lie algebra $se\left(3\right)$ corresponding to the special Euclidean group $SE\left(3\right)$, satisfying: $SE\left(3\right)=\left\{g=\left[\begin{array}{cc} R & p\\ 0_{1\times 3} & 1 \end{array}\right]\in R^{4\times 4}|R\in SO\left(3\right),p\in R^{3}\right\}.$ By the bijection between $R^{3}$ and the Lie algebra $so\left(3\right)$, we can represent $g{\left(s\right)}^{-1}\dot{g}\left(s\right)$ in the 6-dimensional space $R^{6}$. If the twist $V\left(s\right)$ expressed in the parameterized coordinate system $g\left(s\right)$ is obtained, then:

(2) $$V\left(s\right)={\left[v_{x}\;\;v_{y}\;\;v_{z}\;\;u_{x}\;\;u_{y}\;\;u_{z}\right]}^{T}={\left(g{\left(s\right)}^{-1}\dot{g}\left(s\right)\right)}^{\vee }$$

where the first three components of $V\left(s\right)$ represent the 3D linear velocity vector, and the last three components make up the 3D angular velocity vector. The symbol ∨ denotes the map from $se\left(3\right)$ to $R^{6}$. By utilizing Equation (2), the pose data of the spine rod is obtained through Equation (3).

(3) $$\begin{array}{cc} \dot{g}\left(s\right) & =g\left(s\right){\left(V\left(s\right)\right)}^{\wedge }=\left[\begin{array}{cc} \dot{R}\left(s\right) & \dot{p}\left(s\right)\\ 0_{1\times 3} & 0 \end{array}\right]\\ \; & \Rightarrow \left\{\begin{array}{c} \dot{R}\left(s\right)=R\left(s\right){\left(u\left(s\right)\right)}^{\wedge }\\ \dot{p}\left(s\right)=R\left(s\right)v\left(s\right) \end{array}\right. \end{array}$$

where $u\left(s\right)={\left[u_{x}\;\;u_{y}\;\;u_{z}\right]}^{T}$, $v\left(s\right)={\left[v_{x}\;\;v_{y}\;\;v_{z}\right]}^{T}$, and ∧ represents the map from $R^{6}$ to $se\left(3\right)$.

#### 3.1.2. Rigid Connection Segment Model

The rigid connection segment consists of five spacer disks with different hole diameters, fastened together by screws. The length of the rigid connection segment is denoted as $s_{2}$, and its kinematic model can be expressed using (3) as:

(4) $$\begin{array}{cc} p_{rs}\left(s\right) & =p_{ps\_e}\left(s\right)+R_{ps\_e}\left(s\right)e_{3}s_{2}\\ R_{rs}\left(s\right) & =R_{ps\_e}\left(s\right) \end{array}$$

where $p_{ps\_e}\left(s\right)$ and $R_{ps\_e}\left(s\right)$ represent the position and orientation at the start of the rigid connection segment (i.e., the end pose of the proximal segment of the flexible joint), and $e_{3}={\left[\begin{array}{ccc} 0 & 0 & 1 \end{array}\right]}^{T}$ represents the direction along the central axis of the rigid segment in its local coordinate frame.

#### 3.1.3. Kinematics of Rigid Joints

**Figure 2:**
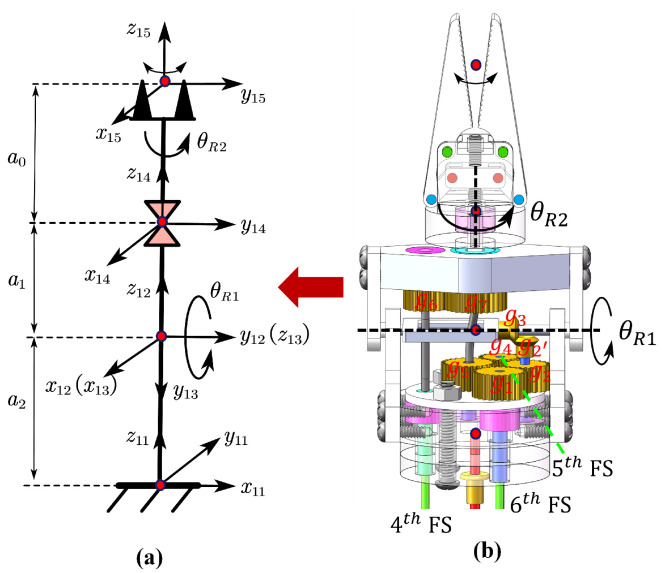
Rigid joint structure and coordinate system definition: (**a**) Establishment of coordinate frames for the rigid joint. (**b**) Structure of the rigid joint.

Based on the end pose data of the distal segment of the flexible joint relative to the world coordinate system $\left[\begin{array}{cc} R_{ds\_e}\left(s\right) & p_{ds\_e}\left(s\right) \end{array}\right]$, the kinematic model of the rigid joint is established using the modified D-H method. The rigid joint coordinate system is defined as shown in Figure 2a, and the distal coordinate system of the flexible joint is defined as $x_{11}y_{11}z_{11}$, where $a_{0}=13.63\;mm$, $a_{1}=14.7\;mm$, and $a_{2}=7.36\;mm$. The modified D-H parameters for the rigid joint are summarized in Table 1. In this case, the gripper end pose data can be obtained using (5).

(5) T150=T110·T1211·T1412·T1514=Rds_espds_es01·s2c200−c1c2c1s2−s1−s1(a0+a1)−c2s1s1s2c1a2+a0c1+a1c10001

To further describe the mapping relationship from the driving space to the joint space for the rigid joint, as shown in Figure 2b, the pitch movement is transmitted through the spur gear pair $g_{1}$, $g_{2}$ and the helical gear pair $g_{2^{\prime }}$, $g_{3}$. The fourth flexible shaft drives the spur gear pair $g_{6}$, $g_{7}$ via a spring to achieve the gripper’s rotational movement, and the fifth flexible shaft drives the spur gear pair $g_{4}$, $g_{5}$ via a spring, which in turn drives the lead screw pair to achieve the gripper’s opening and closing motion. When the sixth flexible shaft rotates by an input angle $\theta_{6}^{in}$, the pitch joint angle $\theta_{R1}$ can be expressed as:

(6) $$\theta_{R1}=\frac{\theta_{6}^{in}}{i_{g_{1}g_{2}}i_{g_{2^{\prime }}g_{3}}}$$

where $\theta_{6}^{in}$ is the input rotation angle of sixth flexible shaft, and $i_{g_{1}g_{2}}i_{g_{2^{\prime }}g_{3}}$ represents the transmission ratio of the gear system, i.e., $\frac{z_{g_{2}}}{z_{g_{1}}}\;\cdot \;\frac{z_{g_{3}}}{z_{g_{2^{\prime }}}}$, where $z_{g_{x}}$ denotes the number of teeth on gear $g_{x}$. Likewise, the rotational joint angle $\theta_{R2}$ can be expressed as:

(7) $$\theta_{R2}=\frac{\theta_{4}^{in}-\Delta \theta_{4}^{s}}{i_{g_{6}g_{7}}}$$

where $\theta_{4}^{in}$ is the input rotation angle of the fourth flexible shaft, and $\Delta \theta_{4}^{s}$ is the torsion angle generated by the spring fixed to fourth flexible shaft.

Since the lead screw for the gripper’s opening and closing motion has a pitch of 0.25 mm and a single-start thread, the mapping relationship between the input rotation angle $\theta_{5}^{in}$ of the fifth flexible shaft and the movement distance $d_{screw}$ of the nut in the lead screw pair is:

(8) $$d_{screw}=\frac{0.25}{2\pi }\left(\frac{\theta_{5}^{in}}{i_{g_{4}g_{5}}}-\Delta \theta_{5}^{s}\right)$$

where $\Delta \theta_{5}^{s}$ is the torsion angle generated by the spring fixed to the spur gear $g_{5}$.

**Figure 3:**
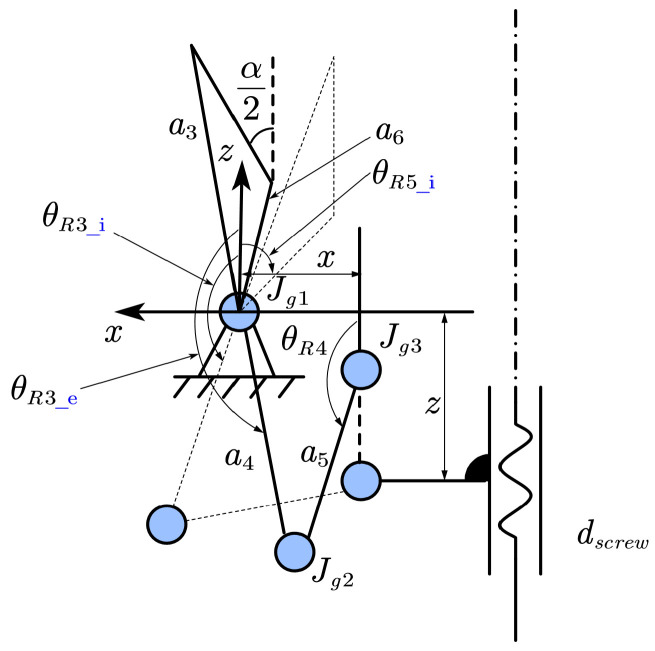
Kinematic diagram of the unilateral gripper mechanism.

Furthermore, a geometric model is established between the nut displacement $d_{screw}$ and the gripper opening angle α. Since the left and right parts of the gripper structure are identical and symmetric, a kinematic diagram of the unilateral gripper mechanism is established, as shown in Figure 3, where the dashed line represents the initial state. The lengths of the linkages are $a_{3}=13.2$, $a_{4}=5.1$, $a_{5}=3$, and $a_{6}=3.4$ mm. The initial angles between linkages $a_{4}$, $a_{6}$, and the *z*-axis are $\theta_{R3\_i}=154.4^{\circ }$ and $\theta_{R5\_i}=-54.4^{\circ }$, respectively. The distance of joint $J_{g_{1}}$ along the *x*-axis is 0.5 mm, while the distance of joint $J_{g_{3}}$ along the *z*-axis is $\left(z_{max}-d_{screw}\right)\in \left[2.1,3.3\right]$ mm. Here, the maximum distance $z_{max}=3.3$ mm occurs when the gripper is closed. Thus, the position of the joint $J_{g_{2}}$ in the *x* and *z* directions can be written as:

(9) $$a_{4}\sin\theta_{R3\_e}=a_{5}\sin\theta_{R4}-x$$

(10) $$a_{4}\cos\theta_{R3\_e}=a_{5}\cos\theta_{R4}-\left(z_{max}-d_{screw}\right)$$

By combining (9) and (10), $\theta_{R3\_e}$ and $\theta_{R4}$ can be obtained. Further, the gripper opening angle α is given by:

(11) $$\alpha =2\mathrm{atan}\left\{\frac{a_{3}\sin\left(\theta_{R3\_e}-\pi \right)-a_{6}\sin\left[\theta_{R5\_i}-\left(\theta_{R3\_e}-\theta_{R3\_i}\right)\right]}{a_{3}\cos\left(\theta_{R3\_e}-\pi \right)-a_{6}\cos\left[\theta_{R5\_i}-\left(\theta_{R3\_e}-\theta_{R3\_i}\right)\right]}\right\}$$

### 3.2. Kinetostatic Model of the Spinal Rods

**Figure 4:**
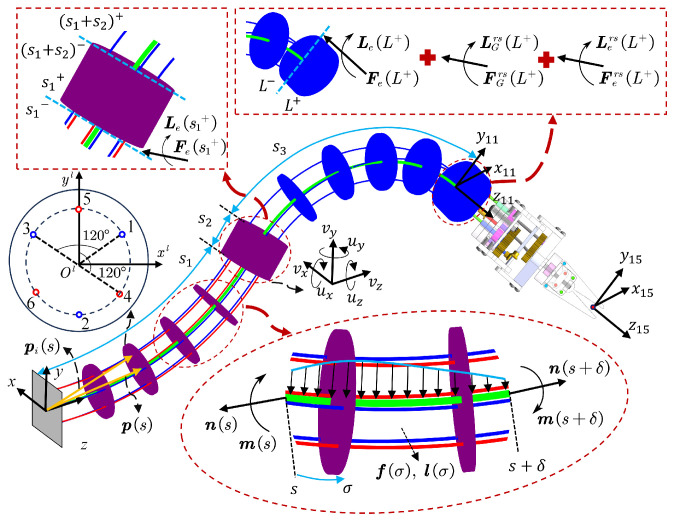
Kinetostatic analysis of bio-inspired rigid-flexible continuum robot.

The kinetostatic analysis of a bio-inspired rigid–flexible continuum robot is shown in Figure 4. Consider any segment of the spinal rod [s,s+δ]. Using the classical equilibrium equations of the Cosserat rod [39], the internal force and internal moment equilibrium equations at the two ends of this segment can be expressed as:

(12) $$\begin{array}{cc} n\left(s\right)-n\left(s+\delta \right)+\int_{s}^{s+\delta }f\left(\sigma \right)d\sigma & =0\\ m\left(s+\delta \right)-m\left(s\right)+p\left(s+\delta \right)\times n\left(s+\delta \right)-p\left(s\right)\times n\left(s\right) & +\int_{s}^{s+\delta }\left(p\left(\sigma \right)\times f\left(\sigma \right)+l\left(\sigma \right)\right)d\sigma =0 \end{array}$$

where $f\left(\sigma \right)$ and $l\left(\sigma \right)$ represent the external force and moment distributions per unit length of the rod.

Next, applying the derivation theorem of integral upper limit function to solve (12) with respect to s+δ, the differential equilibrium equation of the spinal rod is given by:

(13) $$\begin{array}{cc} \dot{n}\left(s+\delta \right)+f\left(s+\delta \right) & =0\\ \dot{m}\left(s+\delta \right)+\dot{p}\left(s+\delta \right)\times n\left(s+\delta \right)+l\left(s+\delta \right) & =0 \end{array}$$

Under the condition of small strains, the two independent strain fields of the spinal rod, namely the linear velocity field and the angular velocity field, are related to shear and elongation as well as bending and twisting [37]. Moreover, based on the assumption of linear elastic material, the linear velocity along the rod’s length and the angular velocity around the axis can be mapped to the internal force and moment of the rod using the constitutive stress–strain law in the local coordinate system as shown in (14).

(14) $$\begin{array}{cc} n\left(s\right) & =R\left(s\right)\;K_{SE}\left(s\right)\left(v\left(s\right)-v^{*}\left(s\right)\right)\\ m\left(s\right) & =R\left(s\right)\;K_{BT}\left(s\right)\left(u\left(s\right)-u^{*}\left(s\right)\right) \end{array}$$

where $v^{*}\left(s\right)$ and $u^{*}\left(s\right)$ are the linear and angular velocity vectors of the spinal rod in the unstressed configuration; $K_{SE}$ and $K_{BT}$ are the stiffness matrices related to shear and elongation as well as bending and twisting of the spinal rod.

To obtain the control equations describing the deformation shape of the spinal rod, differentiate (14) with respect to *s*. Simultaneously, combine the kinematics of flexible joints (3) with the internal force and moment equilibrium Equation (13). Thus, the equation can be expressed as:

(15) $$\begin{array}{cc} \dot{R}\left(s\right) & =R\left(s\right){\left(u\left(s\right)\right)}^{\wedge }\\ \dot{p}\left(s\right) & =R\left(s\right)v\left(s\right)\\ \dot{v}\left(s\right) & ={\dot{v}}^{*}\left(s\right)-K_{SE}^{-1}\left(s\right)\left[\left({\left(u\left(s\right)\right)}^{\wedge }K_{SE}\left(s\right)+{\dot{K}}_{SE}\left(s\right)\right)\right.\left.\left(v\left(s\right)-v^{*}\left(s\right)\right)+R{\left(s\right)}^{T}f\left(s\right)\right]\\ \dot{u}\left(s\right) & ={\dot{u}}^{*}\left(s\right)-K_{BT}^{-1}\left(s\right)\left[\left({\left(u\left(s\right)\right)}^{\wedge }K_{BT}\left(s\right)+{\dot{K}}_{BT}\left(s\right)\right)\right.\\ \; & \;\left.\left(u\left(s\right)-u^{*}\left(s\right)\right)+v{\left(s\right)}^{\wedge }\left(K_{SE}\left(s\right)\left(v\left(s\right)-v^{*}\left(s\right)\right)\right)\right.\left.+R{\left(s\right)}^{T}l\left(s\right)\right] \end{array}$$

Once boundary conditions are given, this control equation can be transformed into a boundary value problem (BVP) for solving.

### 3.3. Kinetostatic Model of the Continuum Robot

Based on the aforementioned content, this section constructs the kinetostatic model of the continuum robot. In particular, considering the distributed load exerted by the flexible shafts on the spinal rod, a more comprehensive kinetostatic model for the flexible joint is established. First, the path of the flexible shafts is defined by the wiring path of the spacer disks fixed on the spinal rod, and the kinematics of the flexible shafts is established.

In the local reference frame of the spacer disk, the position vector of the flexible shaft can be expressed as: $r_{i}\left(s\right)={\left[x_{i}\left(s\right)\;y_{i}\left(s\right)\;0\right]}^{T}$, where i=1,2,3 and i=4,5,6 represent the numbering of the flexible shafts of the distal and proximal segments of the flexible joint, respectively, as shown in Figure 4. Given the pose data of the spinal rod along its length in the world coordinate system $\left[\begin{array}{cc} R\left(s\right) & p\left(s\right) \end{array}\right]$, the position vector of the flexible shaft distributed along the circumference can be described as:

(16) $$p_{i}\left(s\right)=R\left(s\right)r_{i}\left(s\right)+p\left(s\right)$$

Next, it is assumed that the internal force $n_{i}\left(s\right)$ of the flexible shaft is aligned with the tangent direction of the spinal rod and has a magnitude equal to the pulling force $\tau_{i}$ of the flexible shaft. Thus, the internal force of the *i*-th flexible shaft can be expressed as:

(17) $$n_{i}\left(s\right)=\tau_{i}\frac{{\dot{p}}_{i}\left(s\right)}{\|{\dot{p}}_{i}\left(s\right)\|}$$

where $\frac{{\dot{p}}_{i}\left(s\right)}{\|{\dot{p}}_{i}\left(s\right)\|}$ represents the unit tangent vector of the *i*th flexible shaft’s path at *s*.

By differentiating the tangent vector ${\dot{p}}_{i}\left(s\right)$ and using the vector triple product identity, the distributed force applied per unit length on the *i*-th flexible shaft can be written as:

(18) $$\begin{array}{cc} f_{i}\left(s\right) & =-\tau_{i}\frac{{\dot{p}}_{i}\left(s\right)\times \left({\ddot{p}}_{i}\left(s\right)\times {\dot{p}}_{i}\left(s\right)\right)}{\|{\dot{p}}_{i}\left(s\right){\|}^{3}}\\ \; & =\tau_{i}\frac{{\left({\left({\dot{p}}_{i}\left(s\right)\right)}^{\wedge }\right)}^{2}}{\|{\dot{p}}_{i}\left(s\right){\|}^{3}}{\ddot{p}}_{i}\left(s\right) \end{array}$$

where $f_{i}\left(s\right)$ includes the gravity of the *i*-th flexible shaft.

Since the total distributed force acting on the flexible shafts is equal in magnitude and opposite in direction to the distributed force $f_{fb}\left(s\right)$ acting on the spinal rod, and the distributed moment $l_{fb}\left(s\right)$ acting on the spinal rod is the sum of the cross products of the distributed force and the lever arm, the load applied by the flexible shafts to the spinal rod can be described as:

(19) $$\begin{array}{cc} f_{fb}\left(s\right) & =-\sum_{i=1}^{n}f_{i}\left(s\right)\\ l_{fb}\left(s\right) & =-\sum_{i=1}^{n}{\left(R\left(s\right)r_{i}\left(s\right)\right)}^{\wedge }f_{i}\left(s\right) \end{array}$$

To further extend the model control Equation (15), the external loads f(s) and l(s) are expressed as the sum of the loads acting on the spinal rod, $f_{fb}\left(s\right)$ and $l_{fb}\left(s\right)$, and the external loads $f_{e}\left(s\right)$ and $l_{e}\left(s\right)$. The weight of the spinal rod is considered as an external load. Furthermore, using Equation (3), the first and second derivatives of the position vector ${\dot{p}}_{i}\left(s\right)$ and ${\ddot{p}}_{i}\left(s\right)$, expressed in the local coordinate system, can be written as:

(20) p˙iLs=us∧ris+r˙is+vsp¨iLs=p˙is+u˙s∧ris+us∧r˙is+r¨is+v˙s

By combining Equations (15), (19) and (20), a set of implicit differential equations linear in $\dot{u}\left(s\right)$ and $\dot{v}\left(s\right)$ can be derived. To convert them into explicit differential equations and simplify the representation, the following variables are defined:

Ais=−τip˙iLs∧2‖Lp˙is‖3,Gis=−Aisris∧ais=Aisus∧p˙iLs+r˙is+r¨isas=∑i=1nais,bs=∑i=1nris∧aisAs=∑i=1nAis,Gs=∑i=1nGis,Hs=∑i=1nris∧Gis

where i=1,2,...,n represents the flexible shaft ID number.

To account for the coupling between the proximal and distal segments of the flexible joints in the bio-inspired rigid–flexible continuum robot, the control equations incorporate the flexible shafts: six located in the proximal segment and three in the distal segment of the flexible joint. Based on the above discussion, the system control equations can be expressed as:

(21) $$\begin{array}{cc} \dot{R}\left(s\right) & =R\left(s\right){\left(u\left(s\right)\right)}^{\wedge }\\ \dot{p}\left(s\right) & =R\left(s\right)v\left(s\right)\\ \left[\begin{array}{c} \dot{v}\left(s\right)\\ \dot{u}\left(s\right) \end{array}\right] & ={\left[\begin{array}{cc} K_{SE}\left(s\right)+A\left(s\right) & G\left(s\right)\\ G^{T}\left(s\right) & K_{BT}\left(s\right)+H\left(s\right) \end{array}\right]}^{-1}\left[\begin{array}{c} d\left(s\right)\\ c\left(s\right) \end{array}\right] \end{array}$$

where the vectors $c\left(s\right)$ and $d\left(s\right)$ are functions of the state variables $R\left(s\right)$, $p\left(s\right)$, $v\left(s\right)$, and $u\left(s\right)$, expressed as:

$$\begin{array}{cc} c\left(s\right) & =K_{BT}\left(s\right){\dot{u}}^{*}\left(s\right)-\hat{u}{\left(s\right)}^{L}m\left(s\right)-\hat{v}{\left(s\right)}^{L}n\left(s\right)-R{\left(s\right)}^{T}l_{e}\left(s\right)-b\left(s\right)\\ d\left(s\right) & =K_{SE}\left(s\right){\dot{v}}^{*}\left(s\right)-\hat{u}{\left(s\right)}^{L}n\left(s\right)-R{\left(s\right)}^{T}f_{e}\left(s\right)-a\left(s\right) \end{array}$$

where $L_{m}\left(s\right)$ and $L_{n}\left(s\right)$ represent the internal force and moment vectors in the local coordinate system.

For the external load $f_{e}\left(s\right)$ and $l_{e}\left(s\right)$, this paper not only considers the gravity of the unit length spinal rod, but also takes into account the significant weight of the spacer disks made of 316 stainless steel. A mass model for the average mass of the spacer disks is considered. The gravity model for the unit length spinal rod can be described as:

(22) $$f_{e}^{fb}\left(s\right)=\rho_{fb}A_{fb}g\left(s\right)$$

where $\rho_{fb}$ is the material density of the spinal rod, $A_{fb}$ is its cross-sectional area, and the gravity vector $g\left(s\right)={\left[0\;-9.81\;0\right]}^{T}$.

By incorporating the mass of all spacer disks into the material density of the spinal rod, the equivalent material density of the spinal rod can be obtained. The modified gravitational load per unit length of the spinal rod, $f_{e}^{fb}\left(s\right)$, can then be expressed as:

(23) $$f_{e}^{fb}\left(s\right)=\frac{m_{fb}+m_{disk}}{L}A_{fb}g\left(s\right)$$

where $m_{fb}$ is the mass of the spinal rod of the two-segment flexible joint, and *L* is the total length of the manipulator.

A rigid joint is bolted to the distal end of the flexible joint in the bio-inspired rigid–flexible continuum robot. The gravitational effect of this rigid joint is modeled as an equivalent concentrated force and moment applied at this distal end, contributing to the external load. Its equivalent force and moment are given by:

(24) $$\begin{array}{cc} F_{G}^{rs}\left(s\right) & =m_{rs}g\left(s\right)\\ L_{G}^{rs}\left(s\right) & ={\left(r_{c}\left(s\right)\right)}^{\wedge }F_{G}^{rs}\left(s\right) \end{array}$$

where $m_{rs}$ is the mass of the rigid joint, and $r_{c}\left(s\right)$ is the position vector of the center of mass of the rigid joint relative to the end of the flexible joint.

To convert the kinetostatic ODE system in (21) to a BVP, the boundary conditions for the initial position, rigid connection segment, and end position of the bio-inspired rigid–flexible continuum robot are defined as follows. At the initial position, the boundary conditions are specified by (25). Considering that the mechanical boundary conditions are related to the system characteristics and the solution strategy of the control equations, this part will be elaborated in subsequent sections.

(25) $$R\left(0\right)=R_{0}=I_{3\times 3},p\left(0\right)=0_{3\times 1}$$

For the rigid connection segment starting at $s=s_{1}$, if a force $F_{e}\left(s_{1}^{+}\right)$ and a moment $L_{e}\left(s_{1}^{+}\right)$ are acting at this interface (represented by $s_{1}^{+}$), the mechanical equilibrium equations can be written as:

(26) $$\begin{array}{cc} \; & n\left({s_{1}}^{-}\right)=n\left({s_{1}}^{+}\right)+F_{e}\left({s_{1}}^{+}\right)\\ \; & m\left({s_{1}}^{-}\right)=m\left({s_{1}}^{+}\right)+L_{e}\left({s_{1}}^{+}\right)\\ \; & n\left({\left(s_{1}+s_{2}\right)}^{-}\right)=n\left({\left(s_{1}+s_{2}\right)}^{+}\right)\\ \; & m\left({\left(s_{1}+s_{2}\right)}^{-}\right)=m\left({\left(s_{1}+s_{2}\right)}^{+}\right) \end{array}$$

where ${s_{1}}^{-}$ and ${s_{1}}^{+}$ represent the positions before and after $s=s_{1}$, respectively.

It is important to note that the rigid connection segment is made rigid by fastening with screws, and its geometric continuity can be described as:

(27) $$\begin{array}{cc} \; & p\left({s_{1}}^{-}\right)=p\left({s_{1}}^{+}\right)\\ \; & p\left({\left(s_{1}+s_{2}\right)}^{-}\right)=p\left({\left(s_{1}+s_{2}\right)}^{+}\right)\\ \; & R\left({s_{1}}^{-}\right)=R\left({s_{1}}^{+}\right)=R\left({\left(s_{1}+s_{2}\right)}^{-}\right)=R\left({\left(s_{1}+s_{2}\right)}^{+}\right) \end{array}$$

For the distal end of the flexible joint (s=L), considering the gravity of the rigid joint and the external load acting on the rigid joint, as well as the application of external tip force $F_{e}\left(L^{+}\right)$ and tip moment $L_{e}\left(L^{+}\right)$ at $s=s_{3}$ to the spinal rod, the boundary conditions for the internal force and moment at this point are as follows:

(28) $$\begin{array}{cc} n\left(L^{-}\right) & =n\left(L^{+}\right)+F_{e}\left(L^{+}\right)+F_{G}^{rs}\left(L^{+}\right)+F_{e}^{rs}\left(L^{+}\right)\\ m\left(L^{-}\right) & =m\left(L^{+}\right)+L_{e}\left(L^{+}\right)+L_{G}^{rs}\left(L^{+}\right)+L_{e}^{rs}\left(L^{+}\right) \end{array}$$

where $L=s_{1}+s_{2}+s_{3}$. The terms $F_{e}^{rs}\left(L^{+}\right)$ and $L_{e}^{rs}\left(L^{+}\right)$ represent the equivalent external force and moment, respectively, exerted by the subsequent rigid joint, evaluated just after the interface (at $s=L^{+}$). Additionally, the geometric continuity conditions at the interface s=L can be written as:

(29) $$\begin{array}{c} p\left(L^{-}\right)=p\left(L^{+}\right)\\ R\left(L^{-}\right)=R\left(L^{+}\right) \end{array}$$

### 3.4. The Guiding Tubes Model

In this paper, the flexible shaft, which is fixed to the motor output, passes through a specially shaped guiding tube to reach the transition plate. Due to factors such as friction and bending forces introduced by the guiding tube, the driving force $\tau_{i}$ of the flexible shaft often differs from the input driving force $\tau_{i}^{in}$ measured by the force sensor located at the motor output end in the control Equation (21). Additionally, given that the guiding tube has an inner diameter of 3 mm while the flexible shaft diameter is only 1 mm, this paper introduces a model that considers the unilateral friction effect using the capstan equation [40] to describe the force mapping relationship between the two.

(30) $$\begin{array}{c} \tau_{i}=\left(\tau_{i}^{in}+f_{is}\right)e^{\mu_{i}\left(\sum \phi_{i}\right)} \end{array}$$

where $f_{is}$ is the static friction force of the *i*th flexible shaft in its guiding tube, $\mu_{i}$ is the friction coefficient between the *i*th flexible shaft and its guiding tube, and $\phi_{i}$ is the bending angle of the guiding tube through the *i*th flexible shaft.

### 3.5. Numerical Implementation

In this section, the BVP problem for the kinetostatic model of the bio-inspired rigid–flexible continuum robot is solved using the shooting method. The main idea is to transform the BVP into an initial value problem. By guessing the initial conditions, such as the internal force n(0) and internal torque m(0), the ODE system described by Equation (21) is solved using numerical integration. The guessed values are then adjusted to minimize terminal conditions, such as force error $\Delta F_{tip}$ and torque error $\Delta L_{tip}$. In this approach, the proximal and distal segments of the flexible joint are discretized into $N_{1}$ and $N_{2}$ nodes, with a step size of $5\times 10^{-4}$ m.

The shooting method starts the search from initial conditions where all values are zero vectors, i.e., $n\left(0\right)=m\left(0\right)=0_{3\times 1}$. Using the constitutive laws in Equation (14), the initial values of the state variables v(0) and u(0) can be obtained. Since the spinal rod undergoes translational motion and the shear and elongation in the transverse plane (in the *x* and *y* directions) are negligible, the main motion occurs along the length. Thus, the velocity vector for the proximal and distal segments of the flexible joint in the unstressed configuration can be written as: $v_{pro}^{*}\left(0\right)=v_{dis}^{*}\left(0\right)={\left[\begin{array}{ccc} 0 & 0 & 1 \end{array}\right]}^{T}$. At the same time, since the spinal rod has no torsional motion in the unstressed configuration, the angular velocity vector is $u_{pro}^{*}\left(0\right)=u_{dis}^{*}\left(0\right)=0_{3\times 1}$. The initial state variables for the proximal segment of the flexible joint, $v_{pro}\left(0\right)$ and $u_{pro}\left(0\right)$, can then be written as:

(31) $$\begin{array}{cc} v_{pro}\left(0\right) & =K_{SE}^{-1}n\left(0\right)+v_{pro}^{*}\left(0\right)\\ u_{pro}\left(0\right) & =K_{BT}^{-1}m\left(0\right)+u_{pro}^{*}\left(0\right) \end{array}$$

Since the material properties along the spinal rod direction are assumed to be constant, the stiffness matrices $K_{SE}\left(s\right)$ and $K_{BT}\left(s\right)$ are treated as constants.

Using the explicit Runge–Kutta method ode45 in Matlab, the ODE system at $N_{1}+N_{2}$ nodes is solved. To avoid the matrix degradation issue caused by truncation errors in the rotation matrix during numerical integration, where R(s)∉SO(3), the rotation matrix R(s) is converted to an equivalent unit quaternion form $r\left(s\right)={\left[\;r_{0}\left(s\right)\;\;r_{1}\left(s\right)\;\;r_{2}\left(s\right)\;\;r_{3}\left(s\right)\;\right]}^{T}$. The differential equation describing this is given by:

(32) $$\dot{r}\left(s\right)=\frac{1}{2}\Omega \left(u\left(s\right)\right)r\left(s\right)$$

where $u\left(s\right)={\left[\;u_{x}\;\;u_{y}\;\;u_{z}\;\right]}^{T}\in R^{3}$ represents the angular velocity vector in the local coordinate system, and the quaternion mapping matrix $\Omega \left(\;\cdot \;\right):R^{3}\to R^{4\times 4}$ is defined as:

$$\Omega \left(u\left(s\right)\right)=\left[\begin{array}{cccc} 0 & -u_{x} & -u_{y} & -u_{z}\\ u_{x} & 0 & -u_{z} & u_{y}\\ u_{y} & u_{z} & 0 & -u_{x}\\ u_{z} & -u_{y} & u_{x} & 0 \end{array}\right]$$

Next, the fsolve function in Matlab, based on the Levenberg–Marquardt algorithm, is used to solve the BVP problem. The residual vector consists of the terminal force and torque equilibrium errors. The residual vector at the tip of the proximal segment of the flexible joint, considering the point force and point torque applied by the flexible shaft at the attachment point, is defined as:

(33) ΔFtipN1=Fepro(s)−n(s)−∑i=46τip˙iL(s)p˙iL(s)ΔLtipN1=Lepro(s)−m(s)−∑i=46ri(s)∧τip˙iL(s)p˙iL(s)

where *s* is the position at the $N_{1}$-th node of the proximal segment’s end, $F_{e}^{pro}\left(s\right)$ and $L_{e}^{pro}\left(s\right)$ are the external loads at the end of the proximal segment, and i=4,5,6 corresponds to the flexible shaft numbers of the proximal segment.

To account for the coupling effect between the proximal and distal segments of the flexible joint, the residual vector at the tip of the proximal segment is taken as the initial internal force and internal torque vector at the distal segment, i.e.,

(34) $$\begin{array}{cc} v_{dis}\left(0\right) & =-K_{SE}^{-1}\Delta F_{tip}^{N_{1}}+v_{dis}^{*}\left(0\right)\\ u_{dis}\left(0\right) & =-K_{BT}^{-1}\Delta L_{tip}^{N_{1}}+u_{dis}^{*}\left(0\right) \end{array}$$

Similar to Equation (33), the residual vector at the tip of the distal segment of the bio-inspired rigid–flexible continuum robot is given by:

(35) ΔFtipN2=Fedis(s)−n(s)−∑i=13τip˙iL(s)p˙iL(s)ΔLtipN2=Ledis(s)−m(s)−∑i=13ri(s)∧τip˙iL(s)p˙iL(s)

where *s* is the position at $N_{2}$-th node at the end of the distal segment of the flexible joint, $F_{e}^{dis}\left(s\right)$ and $L_{e}^{dis}\left(s\right)$ are the external loads at the end of the distal segment, including the external tip load and rigid joint load, and i=1,2,3 denotes the numbering of the flexible shafts in the distal segment.

The vector $e={\left[\Delta F_{tip}^{N_{2}}\;\;\Delta L_{tip}^{N_{2}}\right]}^{T}$ is used as the residual vector returned by the Levenberg–Marquardt algorithm. The optimal initial values $n^{*}\left(0\right)$ and $m^{*}\left(0\right)$, which satisfy the terminal equilibrium conditions, are obtained by minimizing the residual vector:

(36) $${\left[n^{*}\left(0\right)\;\;m^{*}\left(0\right)\right]}^{T}=\underset{n(0),m(0)}{\arg\min}e\left(n(0),m(0)\right)$$

## 4. Experimental Evaluation

### 4.1. Prototype and Experiment Setup

**Figure 5:**
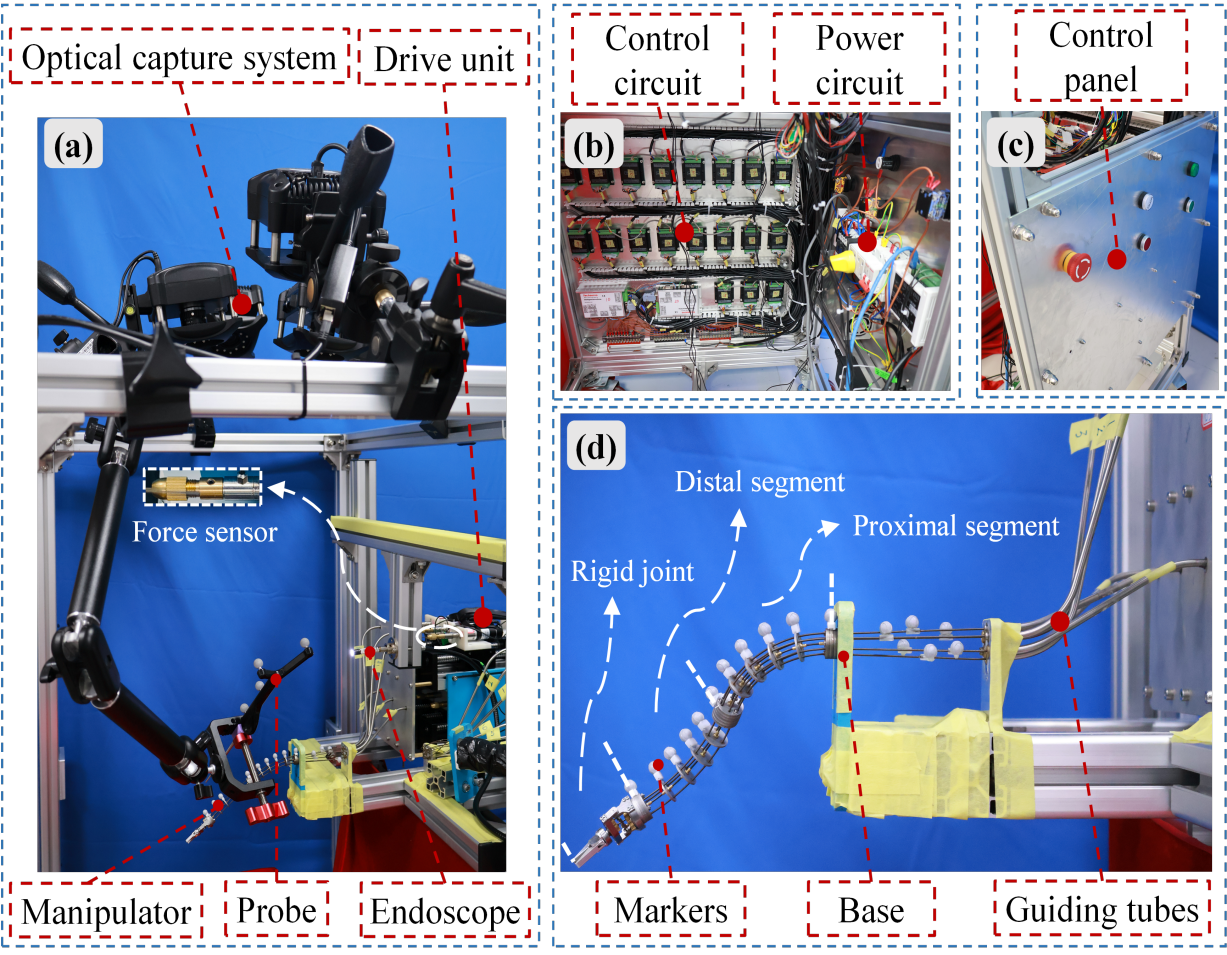
Prototype experimental system of the bio-inspired rigid–flexible continuum robot: (**a**) Overview of the experimental setup. (**b**) Physical layout of the control and power circuits. (**c**) Control panel for system operation. (**d**) Physical prototype of the manipulator.

To verify the kinetostatic model of the bio-inspired rigid–flexible continuum robot, a prototype experimental system was built, as shown in Figure 5. The system mainly includes: power circuits, control circuits, an infrared motion capture system, drive units, a manipulator, and an endoscope. To measure the position data of the manipulator in space, a NOKOV optical 3D motion capture system was used, consisting of four infrared motion capture cameras (NOKOV, Beijing, China), one switch (ONV, Shenzhen, China), reflective markers, and a motion capture workstation. The motion capture cameras can achieve a maximum resolution of 1280 × 1024 with a frame rate of 240 FPS, and the 3D precision is ±0.2 mm. A handheld probe (NOKOV, Beijing, China) is connected to the motion capture system via a linkage bracket for precise measurement of small-scale structures. In the drive unit, three servo motors (Techrobots, Shenzhen, China) provide torsional driving force for the rigid joints, while six servo motors drive the flexible joints through tensile force. To obtain the tensile force data of the flexible shafts, six force sensors (Ryder, Shenzhen, China) are fixed to the motor output shafts through the intermediate modules. Reflective markers with a diameter of 6 mm are attached to the top of the manipulator’s spacer disks, with a total of 11 markers used to capture the manipulator’s shape data during the experiment.

The overall control system operates as follows: Upon pressing the button on the control panel, the system is powered on. The power circuit supplies energy to the drive units and converts 220V AC to 24V DC needed for the control circuits. The upper computer, equipped with a graphical user interface (GUI) developed using RAD Studio 11.3, serves as the supervisory control platform. Through the drive units, the upper computer commands the servo motors to operate in velocity mode. During experiments, force sensors continuously monitor the tensile force on the flexible shafts. When the measured force reaches a predefined threshold, the control system automatically halts the corresponding motor to prevent overloading. At this point, the infrared motion capture system is triggered to record the spatial position data of the reflective markers attached to the manipulator.

### 4.2. Parameter Calibration

**Figure 6:**
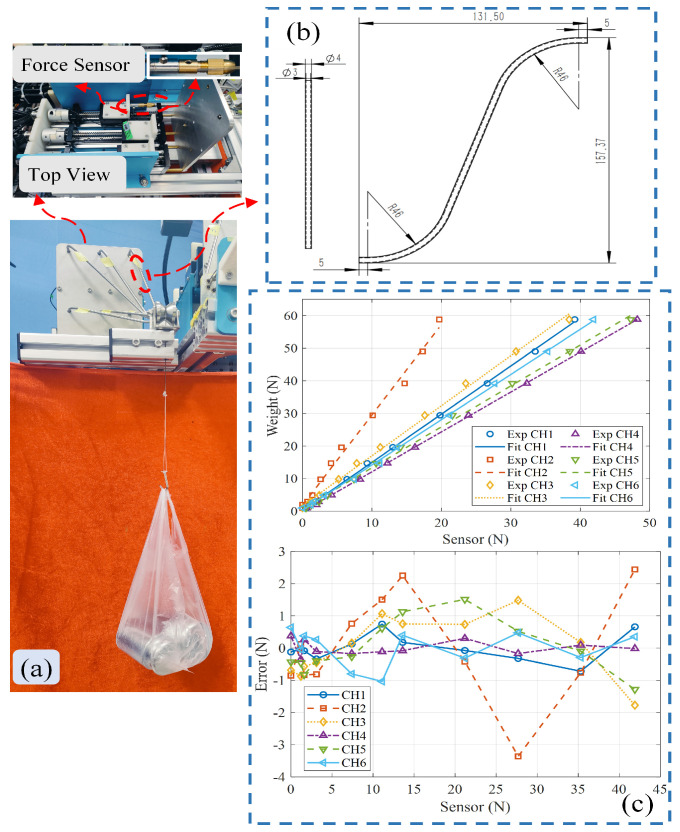
Parameter calibration of the guide tube: (**a**) Experimental setup for guide tube parameter calibration. (**b**) Design drawing of the guide tube for the first flexible shaft. (**c**) Calibration results of guide tube parameters.

This section mainly performs the calibration of the guide tube parameters and the mechanical parameter calibration experiment for the flexible shafts and spine rods. Figure 6b shows the 2D dimension drawing of the guide tube for the first flexible shaft, which includes two curved segments with a bending angle of 46° and one straight connecting segment. The other guide tubes are also made up of two curved segments with the same bending angle and one straight segment, with bending angles $\phi_{i}={\left[46^{\circ },46^{\circ },46^{\circ },46^{\circ },46^{\circ },33^{\circ },38^{\circ }\right]}^{T}$. To obtain the static friction force $f_{is}$ and the friction coefficient $\mu_{i}$ in (30), a guide tube parameter calibration setup was designed, as shown in Figure 6a, where the flexible shaft driving force $\tau_{i}$ is replaced by standard mass weights, and the driving force $\tau_{i}^{in}$ is measured by force sensors. First, 11 sets of standard masses, ranging from 100 g to 6000 g, were used in the experiment. Then, using the 11 sets of driving force variation data collected in the experiment, the static friction force $f_{is}$ and friction coefficient $\mu_{i}$ were obtained by least squares fitting. Since the dimensions of the guide tubes for the six flexible shafts are not the same, a total of 66 experiments were conducted, with the calibration results shown in Figure 6c. The static friction forces $f_{is}$ and friction coefficients $\mu_{i}$ for the six guide tubes are: $f_{is}={\left[0.146\;N,0.664\;N,1.088\;N,-0.105\;N,0.625\;N,0.247\;N\right]}^{T}$, and $\mu_{i}={\left[0.243,0.635,0.266,0.125,0.197,0.247\right]}^{T}$.

**Figure 7:**
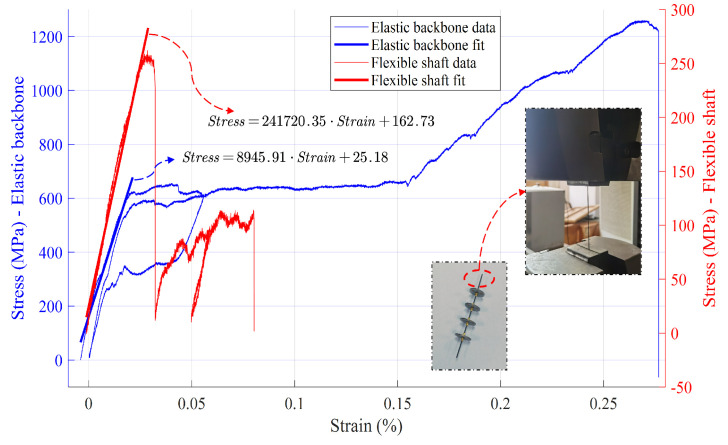
Tensile experiment for determining mechanical parameters of the flexible shaft and spine rod.

To determine the mechanical parameters of the flexible shafts and spine rods, a tensile experiment was conducted using an MTS E45.305 model tensile machine, as shown in Figure 7. For the 1 mm spine rod, the YS/T 1147-2016 super-elastic nickel-titanium alloy tensile testing method [41] was used. For the 1 mm driving flexible shaft, the GB/T 228.1-2021 metallic material tensile test method [42] was applied. The gauge length for both was 50 mm, with a distance between the grips of 170 mm, and a stretching rate of 1 mm min^−1^. The stress–strain curves are shown in Figure 7. The stress–strain curves for both the spine rod and flexible shaft in the elastic range were fitted, and their Young’s moduli were found to be 24,172.35 MPa and 8945.91 MPa, respectively, with Poisson’s ratios of 0.3 and 0.32.

### 4.3. The Effect of Spacer Disks on Flexible Joints

**Figure 8:**
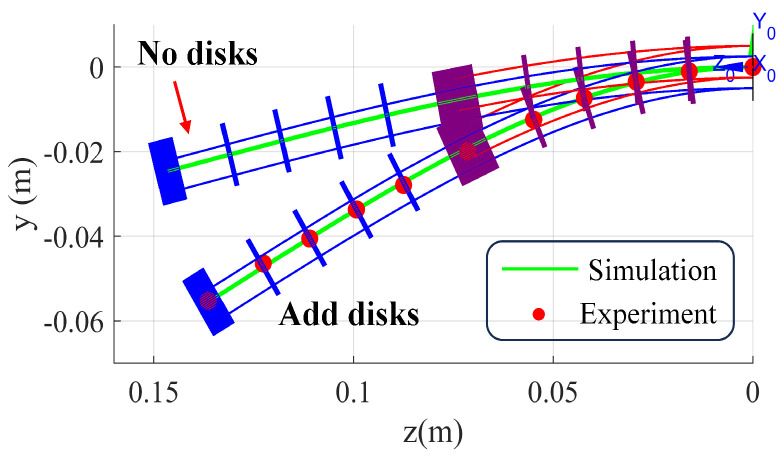
Simulation and experimental validation of the bio-inspired rigid–flexible continuum robot under zero input/zero load conditions.

Figure 8 shows the simulation and experimental data of the bio-inspired rigid–flexible continuum robot under zero input and zero load conditions. The red solid circles represent the 3D position data of the reflective markers measured by the infrared motion capture system, while the green solid line shows the position information of the spinal rod obtained from simulation. In this study, the position data of each reflective marker were subtracted by the position data of the reflective marker placed on the base, aligning the coordinate systems of the infrared motion capture system and the bio-inspired rigid–flexible continuum robot. Without considering the gravitational effect of the spacer disks, the actual external load in the static model of the continuum robot is only the body force from the spinal rod. In this case, the maximum error and average error between the simulation and experimental data are 30.6 mm and 20.56% of the manipulator length, respectively. However, when the gravitational effect of the spacer disks is included in the static model, the maximum error and average error between the simulation and experimental data are reduced to 0.60% and 0.25% of the manipulator length, respectively.

### 4.4. Shape Perception of Continuum Robot

**Figure 9:**
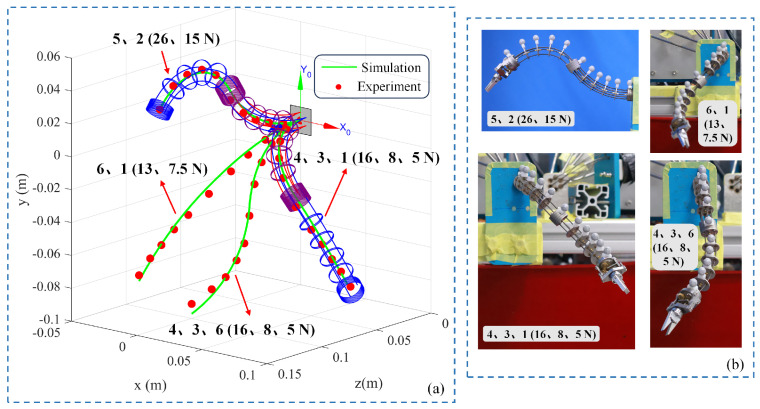
Shape perception and static morphology of the bio-inspired rigid–flexible continuum robot under antagonistic drive: (**a**) 3D shape sensing results of the hybrid rigid–flexible robot under different antagonistic driving modes. (**b**) Static illustrations of the hybrid rigid–flexible robot in various drive configurations.

Figure 9a shows the 3D shape sensing data of the bio-inspired rigid–flexible continuum robot both in-plane and out-of-plane, including antagonistic driving with two and three flexible shafts. Figure 9b shows static illustrations of the bio-inspired rigid–flexible continuum robot under various drive configurations. For the in-plane configuration of the manipulator, when 26 N and 15 N driving forces are applied to the fifth and second flexible shafts, the maximum error and average error are 5.7 mm and 1.6 mm, accounting for 3.84% and 1.05% of the manipulator length, respectively. When 13 N and 7.5 N driving forces are applied to the sixth and first flexible shafts, the maximum error and average error are 4.7 mm and 2.4 mm, accounting for 3.18% and 1.63% of the manipulator length, respectively. For the out-of-plane configuration of the manipulator, when 16 N, 8 N, and 5 N driving forces are applied to the fourth, third and sixth flexible shafts, the maximum error and average error are 6.0 mm and 3.0 mm, accounting for 4.03% and 2.01% of the manipulator length, respectively. When 16 N, 8 N, and 5 N driving forces are applied to the fourth, third and first flexible shafts, the maximum error and average error are 5.9 mm and 2.4 mm, accounting for 3.96% and 1.63% of the manipulator length, respectively. The quantitative analysis indicates that the proposed kinetostatic model is reliable for the shape perception of the bio-inspired rigid–flexible continuum robot.

**Figure 10:**
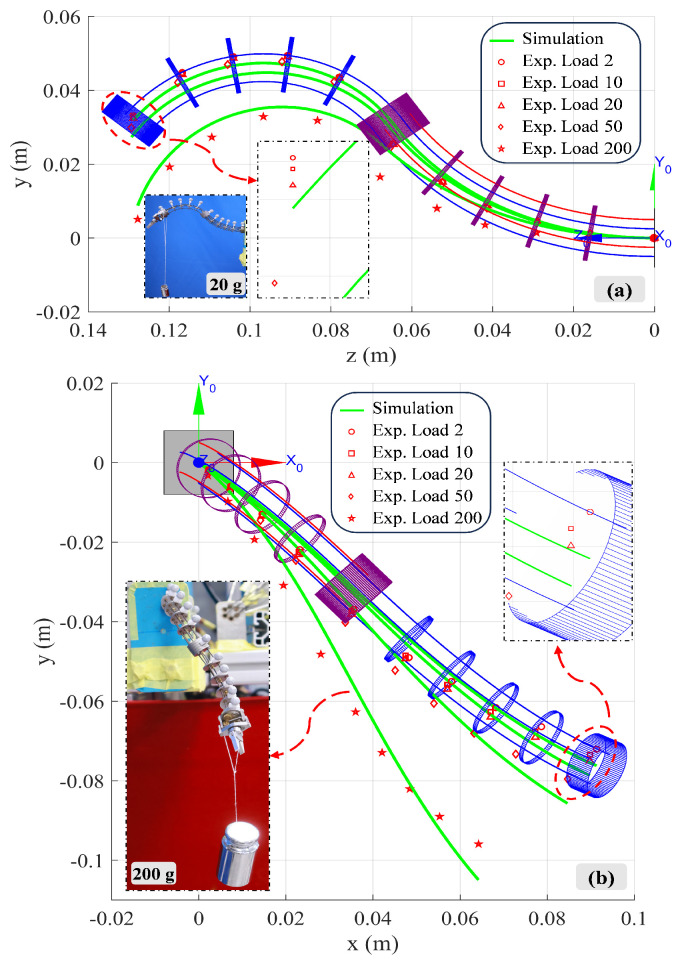
Loading experiments on the bio-inspired rigid–flexible continuum robot: Comparison between model prediction and measured data: (**a**) In-plane drive: Effect of tip load on deformation and model error (flexible shafts 5/2 driven). (**b**) Out-of-plane drive: Effect of tip load on deformation and model error (flexible shafts 4/3/1 driven).

Further, the proposed kinetostatic model was evaluated on the bio-inspired rigid–flexible continuum robot with known tip loads, as shown in Figure 10. The continuum robot underwent load experiments with two driving force configurations, and standard masses of 2 g, 10 g, 20 g, 50 g, and 200 g were used as tip loads. Figure 10a shows the results when 26 N and 15 N in-plane driving forces were applied to the fifth and second flexible shafts. It can be seen that when the tip load is less than 50 g, the manipulator remains rigid with a deformation of only 3.3 mm in the *y*-axis direction. The maximum error and average error are 5.6 mm and 2.0 mm, accounting for 3.74% and 1.33% of the manipulator length, respectively. When the tip load is 200 g, the average error increases to 3.9 mm, accounting for 2.61% of the manipulator length. Figure 10b shows the results when 16 N, 8 N, and 5 N out-of-plane driving forces were applied to the fourth, third and first flexible shafts. When the tip load is less than 50 g, the manipulator still maintains high rigidity with a deformation of only 7.6 mm in the *y*-axis direction. The maximum error and average error are 6.1 mm and 2.6 mm, accounting for 4.1% and 1.71% of the manipulator length, respectively. When the tip load is 200 g, the average error increases to 4.5 mm, accounting for 3.03% of the manipulator length. In summary, the proposed model has been proven to accurately capture the shape of the manipulator under inter-segment interactions and known external loads.

### 4.5. Rigid Joint Motion Performance

**Figure 11:**
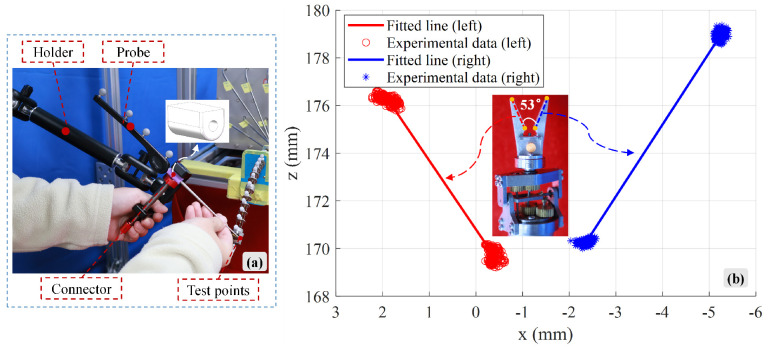
Rigid joint gripper: Maximum opening angle analysis: (**a**) Motion capture data acquisition system with probe stabilization fixture. (**b**) Visualization of processed position data in the xoz plane.

For the rigid joints in the bio-inspired rigid–flexible continuum robot, functional evaluations were conducted for the gripper’s opening and closing, rotation, and pitching motions. The driving force of the second flexible shaft is transmitted to the gripper structure via a screw pair mechanism. To accurately obtain spatial coordinate information at specific positions of the small-sized mechanism, a handheld motion capture probe was used. To address the measurement errors caused by hand tremors, a specialized connecting device was designed to fix the probe onto a universal adjustable linkage bracket. The data acquisition process is shown in Figure 11a. To determine the maximum opening angle of the gripper, the probe tip was brought into contact with the tip and end points of the left and right grippers, as indicated by the orange solid circles in Figure 11b. Subsequently, 200 frames of position data were collected for each sampling point using the Nokov infrared optical motion capture system. To reduce errors from hand tremors, muscle fatigue, and environmental vibrations during the measurement process, outliers based on σ were removed from the data using the central limit theorem. The average values for each sampling point were then calculated, and the angle between the two spatial lines was determined. Figure 11b shows the position data of the spatial lines in the xoz plane. The experimental results showed that the maximum opening angle of the gripper in the rigid joint was 53°.

**Figure 12:**
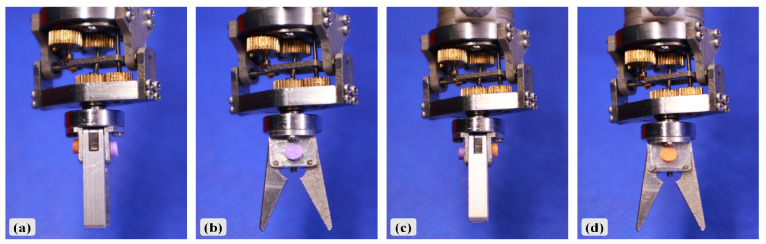
Assessment of gripper rotational function: (**a**) Gripper state at 90° rotation. (**b**) Gripper state at 180° rotation. (**c**) Gripper state at 270° rotation. (**d**) Gripper state at 360° rotation.

In the rotational motion functional test, purple and orange markers were attached to the upper and lower surfaces of the gripper base. The first flexible shaft was driven by a motor to achieve 360° rotation of the gripper. Figure 12 shows the gripper at every 90° interval of rotation.

**Figure 13:**
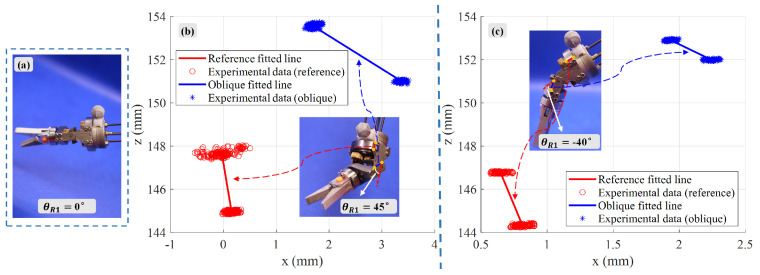
Evaluation of rigid joint pitching motion: (**a**) Initial state ($\theta_{R1}=0^{\circ }$). (**b**) Spatial lines for positive pitch angle calculation (xoz plane). (**c**) Spatial lines for negative pitch angle calculation (xoz plane).

A similar data collection and analysis method, as used in the gripper opening and closing functional evaluation, was applied to the pitching motion functional test of the rigid joint. When the rigid joint was in its initial state, i.e., with a pitching joint angle of $\theta_{R1}=0^{\circ }$, as shown in Figure 13a, the motor drove the third flexible shaft in the forward direction, reaching the maximum positive pitching angle of 45°. When the third flexible shaft was driven in reverse, the maximum negative pitching angle of −40° was reached. Figure 13b and Figure 13c show the spatial lines used to calculate the positive and negative pitching angles in the xoz plane, respectively.

### 4.6. Multi-Obstacle Environment Omnidirectional Bending and Long-Distance Target Reaching

**Figure 14:**
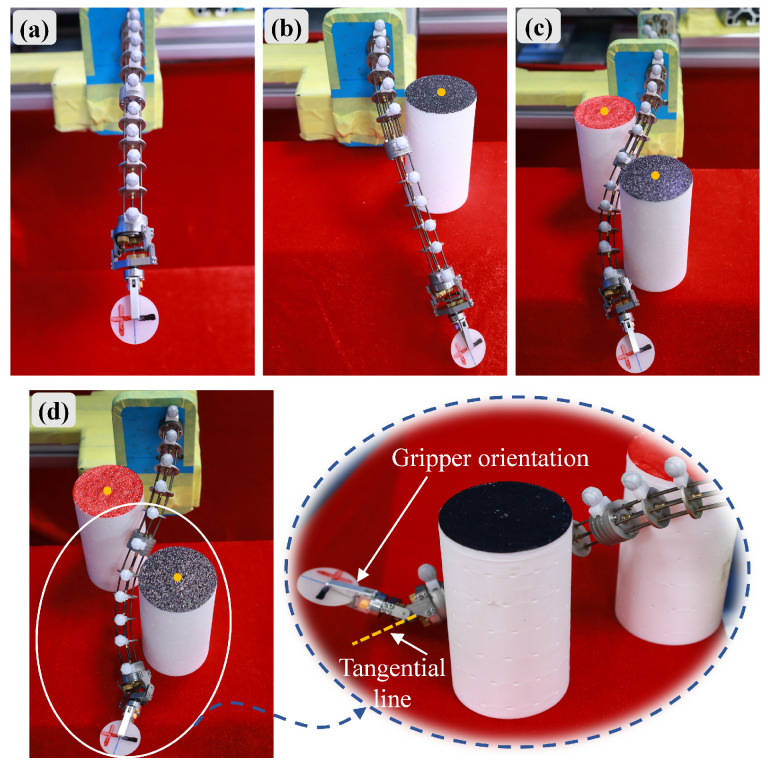
Demonstration of obstacle avoidance and extended reach capabilities: (**a**) Initial robot configuration. (**b**) Single obstacle avoidance using proximal flexible segment. (**c**) Multi-obstacle avoidance using dual flexible segments. (**d**) Extended reach achieved via rigid joint pitching.

To evaluate the effectiveness and flexibility of the flexible shaft tensile–torsional synergistic actuation in the bio-inspired rigid–flexible continuum robot, a multi-obstacle avoidance and long-distance target reaching experiment was designed. The initial state of the manipulator is shown in Figure 14a. First, a single obstacle avoidance experiment was conducted, with only the proximal segment of the flexible joint being driven. The second flexible shaft gripped a 1 mm thick, 22 mm diameter silicone pad. A 41 mm diameter cylindrical obstacle was placed in front of the robot’s right side, with the origin coordinates of the cylindrical black surface being (22.3, −18.8, 41.9) mm, as indicated by the orange solid origin point. The fourth flexible shaft was driven to achieve single obstacle avoidance by the flexible robot, as shown in Figure 14b. Next, a multi-obstacle avoidance experiment was performed with two cylindrical obstacles, each 41 mm in diameter, placed in the left rear and right front of the robotic arm. The origin coordinates of the cylindrical black and red surfaces were (−0.8, −29.2, 98.2) mm and (−33.9, −28.1, 30.3) mm, respectively. In collaboration with the first and sixth flexible shafts and the linear motion module, the bio-inspired rigid–flexible continuum robot gripped the silicone pad and bypassed the red and black obstacles. The final bent configuration of the manipulator is shown in Figure 14c. To reach a further position, the rigid joint was pitched via the positive pitching angle, as shown in Figure 14d, using the first, second and sixth flexible shafts in tensile–torsional synergistic actuation. Based on the above experiments, the effectiveness and high flexibility of the bio-inspired rigid–flexible continuum robot were further validated.

## 5. Discussion

This study designs and verifies a novel bio-inspired rigid-flexible continuum robot driven by flexible shafts tension-torsion synergy, which cleverly utilizes the dual characteristics of the flexible shaft in both tension and torsion. Traditional continuum robot structures often face a trade-off between mechanical complexity and flexibility. In contrast, this study simplifies the robot’s overall structure and achieves higher flexibility by using the flexible shaft both as a medium for tension-driven flexible joint bending and as a power source for transmitting torque to the end rigid joint. Specifically, this research constructs a new type of rigid-flexible hybrid robot mechanism, with six flexible shaft inputs and seven degrees of freedom outputs, combining “dual-segment flexible body + single-segment rigid body” design, and establishes the corresponding kinetostatic model. This innovative structural design provides new insights for achieving more compact and flexible continuum robots. Moreover, the compact structure, flexibility, and precise control afforded by the tension-torsion synergy mechanism suggest significant potential for this continuum robot in practical applications. For instance, in minimally invasive medical procedures, it could navigate and operate effectively within confined and delicate anatomical regions, such as the oropharyngeal cavity, enabling intricate manipulation. Additionally, in industrial inspection, its capabilities are well-suited for accessing complex or restricted spaces to examine and maintain critical components, thereby potentially enhancing inspection efficiency and accuracy. Furthermore, another core contribution of this study is the proposal and experimental verification of a comprehensive kinetostatic model, which is based on Cosserat rod theory and couples the “spinal rod + flexible shaft” effect, while integrating the guide tube friction model and distributed spacer disk gravity model. Experimental evaluation results strongly support the validity and accuracy of the model.

First, the experimental results of the spacer disk gravity effect demonstrate the necessity of including distributed gravity in the model. When the spacer disk gravity was not considered, the maximum deviation between the model prediction and actual measurement shape was as high as 30.6 mm (20.56% of the manipulator’s total length), indicating that considering only the weight of the spinal rod as the actual external load is insufficient. It is worth noting that we initially attempted a discretized gravity model, which applied the gravity produced by the concentrated mass of the spacer disk (about 12.807 N) within the actual length range occupied by the spacer disk (s at the spacer disk position), and considered only the small gravity (about 0.054 N) generated by the spinal rod body in the regions between the spacer disks. Although this discrete model conceptually aligns more closely with the physical structure, simulation tests showed that this stepwise external force function ($f_{e}\left(s\right)$) with a sharply varying distribution along the rod length s caused difficulties in numerical solutions (e.g., ode45). To overcome this challenge and obtain physically reasonable and numerically stable solutions, we ultimately adopted the average mass model presented in this study. This model averages the spacer disk mass along the manipulator’s length, producing a smoother and continuous external force distribution function.

Next, shape perception experiments systematically evaluated the model’s predictive ability under various driving configurations (in-plane/out-of-plane, dual/tri-flexible shafts drive). The experimental results showed that, under no external load conditions, the robot’s three-dimensional shape predicted by the model closely matched the actual shape measured by a high-precision optical motion capture system. Under different driving force combinations, the maximum shape prediction error was controlled within 6.0 mm (approximately 4.03% of the total length), and the average error was less than 3.0 mm (approximately 2.01% of the total length). This fully demonstrates that the dual-segment “spinal rod + flexible shaft” coupling model established based on Cosserat rod theory can effectively capture the complex deformation behavior of the flexible segment under flexible shaft tension-driven actuation. Additionally, load experiments further tested the model’s robustness in the presence of external disturbances. The results showed that even under a 200 g end-load, the model could still predict the robot’s deformation with high accuracy (e.g., under a 200 g in-plane load, the average error was 3.9 mm, accounting for 2.61% of the total length). In summary, the model’s effectiveness was validated, and the structural rigidity characteristics of this rigid–flexible hybrid design were demonstrated within a certain load range.

Although the model demonstrates good predictive accuracy, there is still some deviation between the theoretical predictions and experimental measurements. The potential sources of error can be summarized as follows: First, model simplifications and assumptions: The Cosserat rod model is a simplification of the mechanical behavior of continuous media and does not fully capture the material’s nonlinearity, viscoelasticity, or hysteresis effects. Additionally, using the spacer disk average mass model, as opposed to the ideal discrete spacer disk mass model, introduces approximation errors. Second, parameter calibration errors: The calibration of parameters such as material elastic modulus, Poisson’s ratio, and guide tube friction coefficient inevitably involves measurement and fitting errors. Third, prototype manufacturing and assembly tolerances: The manufacturing precision of the continuum robot prototype, assembly gaps and alignment accuracy of components (e.g., spacer disks, spinal rods, guide tubes), and the initial slackness of the flexible shaft inside the guide tube could lead to deviations in the actual system’s mechanical behavior from the ideal model. Finally, measurement system errors: The sampling accuracy of the optical motion capture system (±0.2 mm), the accuracy of the reflective marker placement, as well as the measurement accuracy and zero drift of force sensors, contribute to inherent measurement errors in the measurements.

## 6. Conclusions

This study designs and verifies a novel bio-inspired rigid–flexible continuum robot driven by flexible shaft tension–torsion synergy, aimed at addressing the trade-off between complexity and flexibility in traditional continuum robot mechanisms. By using the flexible shaft to achieve both tension-driven bending and torque transmission, the structure is simplified while flexibility is enhanced. A “dual-segment flexible body + single-segment rigid body” mechanism with six flexible shaft inputs and 7-DoF outputs is constructed, and the corresponding kinetostatic model is established. Experimental results verify the necessity of including distributed spacer disk gravity in the model. Shape perception experiments show that the model can accurately predict the robot’s shape under different drive configurations, with the maximum error controlled within 6.0 mm (approximately 4.03% of the total length) and the average error less than 3.0 mm (approximately 2.01% of the total length). Load experiments further validate the robustness of the model under external disturbances, maintaining high prediction accuracy even with a 200 g load, demonstrating the structural rigidity of the design.

In future research, we will focus on improving the modeling accuracy and control performance of the bio-inspired rigid–flexible continuum robot driven by flexible shafts tension–torsion synergy. On the one hand, we will conduct in-depth studies on the nonlinear constitutive relationships of flexible materials, constructing more accurate mechanical models, and incorporating model order reduction strategies to reduce computational complexity, thus laying the foundation for real-time control. On the other hand, we will explore advanced model-based control strategies and data-driven control methods, utilizing the model’s predictive capabilities and machine learning techniques to compensate for system dynamics, enabling precise trajectory tracking and force control. This will further overcome the challenges of flexible robot modeling and control, promoting its practical applications in fields such as healthcare and industry.

## Figures and Tables

**Table 1:** Modified D-H parameters for the rigid joint.

Link	$\alpha_{i-1}$	$a_{i-1}$	$d_{i}$	$\theta_{i}$
12	$0^{\circ }$	0	$a_{2}$	$-90^{\circ }$
13	$-90^{\circ }$	0	0	$\theta_{R1}$
14	$90^{\circ }$	0	$a_{1}$	$\theta_{R2}$
15	$0^{\circ }$	0	$a_{0}$	$0^{\circ }$

## Data Availability

The original contributions presented in this study are included in the article. Further inquiries can be directed to the corresponding author.
